# Public/private sector partnership for emerging infections.

**DOI:** 10.3201/eid0707.017707

**Published:** 2001

**Authors:** E C Bond

**Affiliations:** Burroughs Wellcome Fund, Research Triangle Park, North Carolina 27709-3901, USA. qbond@bwfund.org

## Abstract

This paper gives examples of public/private partnerships that support research, support drug development and that advance policy development, suggesting that such partnerships can advance our understanding and control of emerging infections. The investment in emerging infectious diseases from government and from industry is currently much larger than that from philanthropy. Nevertheless philanthropy, even with limited dollars, is able to play a catalytic function and provide risk capitol for innovative partnerships and could in the future play an even larger role if the value of such investment is better defined and argued to recruit additional dollars to this area.


### Public/Private Sector Partnership For Emerging Infections

**Enriqueta C. Bond**
Burroughs Wellcome Fund, Research Triangle Park, North Carolina, USA

This paper gives examples of public/private partnerships that support research, support drug development and that advance policy development, suggesting that such partnerships can advance our understanding and control of emerging infections. The investment in emerging infectious diseases from government and from industry is currently much larger than that from philanthropy. Nevertheless philanthropy, even with limited dollars, is able to play a catalytic function and provide risk capitol for innovative partnerships and could in the future play an even larger role if the value of such investment is better defined and argued to recruit additional dollars to this area.

Emerging infectious diseases cause a disproportionate burden in developing countries, hindering economic and political advancement. Because of global interdependence, modern transportation, trade, and changing social and cultural patterns, emerging or reemerging infections are also threats to the United States (1). Therefore, the United States has a vital and direct stake in the health of people around the world, both out of humanitarian concern and enlightened self-interest.

In the battle against infectious diseases, drugs, vaccines, and pesticides are important weapons. Given the lack of substantial markets for such products in developing countries, public/private partnerships are essential to the development of new ways to prevent and treat infectious diseases in those populations that are poor and where the burden of disease lies, largely in the developing world.

### Kinds of Partnerships

Our understanding and control of emerging infections can be advanced by several types of public/private partnerships, such as those that support research, develop drugs and vaccines, or advance policy development.

The role of philanthropical investments in partnerships that support medical research is also an important consideration. Strong investments in public health agencies, both in facilities and programs, will enable public/private partnerships to reach their full potential.[1]

### Partnership To Support Research: Sequencing the Malaria Genome

Sequencing the genomes of microorganisms, those that either afflict millions of persons primarily in poorer parts of the world or those that afflict persons in both the developing and developed worlds in much smaller numbers, needs champions among public or philanthropic funders of biomedical research because corporations will generally not be interested in research on such organisms.

Sequence information can be essential to identifying therapeutic approaches to diseases such as malaria. In response to the rising global incidence of malaria, the Burroughs Wellcome Fund (BWF) has joined with the Wellcome Trust in the United Kingdom, the U.S. Department of Defense, and the National Institute of Allergy and Infectious Diseases (NIAID) to support the Malaria Genome Project, an international effort to sequence the genetic code of *Plasmodium falciparum*, the major causative agent of malaria. This partnership has supported not only the sequencing of the parasite's 14 chromosomes but also the development of databases and new tools for studying the expression of the newly identified genes. The sequencing is being carried out at the Sanger Center, at The Institute for Genomic Research (TIGR) (with colleagues from the Department of Defense) and at Stanford University. Data from this project already have provided new insights into the parasite's biology and have helped advance vaccine research (2). The malaria research community will participate in a "jamboree," similar to that held by scientists working on *Drosophila*, at the end of 2001 to finish the sequencing of the malaria genome.

The experience of funding this large-scale project has been even more rewarding than BWF had anticipated. Along with the new scientific insight it has provided, the Malaria Genome Project has grown into a model consortium that comprises a collaborative and interactive group able to work with scientists locally and globally. Funders and scientists have developed a strong relationship that allows "just in time" acquisition of resources and an agile, responsive approach to planning. In addition, flexibility in the project's funding (because different organizations can support different aspects) has allowed the consortium to take advantage of new technologies and techniques. For example, the Burroughs Wellcome Fund was able to provide seed dollars to convene the scientific community to

Address for correspondence: Enriqueta C. Bond, Burroughs Wellcome Fund, P.O. Box 13901, RTP, NC 27709-3901, USA; fax: 919-941-5884; e-mail: qbond@bwfund.org

[1] For more information about public/private partnerships, see Roy Widdus' work at www.ippph.org. The site identifies all significant public/private partnerships and their origins, aims, governance structures, modus operandi, degree of success, constraints, and difficulties. The goal of the project is to assist in the creation of new, effective partnerships.

discuss needs, select the strain to be sequenced and provide funding for pilot work. The National Institute of Allergy and Infectious Diseases and the Wellcome Trust provided funds for sequencing while the Department of Defense provided expertise and tool development.

### Partnership for Drug Development: Medicines for Malaria Venture

The Medicines for Malaria Venture (MMV) is a new public/private partnership developed under the umbrella of the World Health Organization's (WHO) Roll Back Malaria Program (Ridley, pers. comm.). Like the Malaria Genome Project, the driving force behind the creation of MMV is the disease burden of malaria in the developing world. The lack of vaccines and increasing problems of drug resistance to the available drugs mean that new antimalarial drugs are urgently needed.

Under the leadership of Win Gutteridge (WHO/TDR), a strategic planning group was assembled from persons representing large pharmaceutical companies, development agencies, foundations, and international health organizations. The planning group confirmed the urgent need for new antimalarial drugs but also noted that the market could not support the high cost and risk associated with pharmaceutical drug company research and development (R&D) (as much as $500 million per drug candidate). Since expertise in pharmaceutical R&D resides with industry, the planning committee sought to combine public and private sector resources and expertise to "lower the risk" of drug development and encourage industry to make new drugs.

MMV is set up as a not-for-profit business with a mission of fostering and financing the discovery and development of new, affordable antimalarial drugs. The organization's goal is to have one new product granted regulatory approval every five years and to make arrangements for the products' commercialization. With each project, appropriate intellectual property would be owned by MMV with commercialization through out-licensing.

To carry out its mission, MMV created a "public venture capital fund" to support R&D projects on a competitive basis. MMV accesses knowledge, experience, gifts-in-kind, and, if appropriate, money from the private sector. However, MMV seeks most of its financial resources from the public and philanthropic sector.

As of May 2000, MMV has been constituted as an independent foundation in Switzerland, the board has been appointed, a CEO selected, the business plan completed, and a portfolio of R&D projects funded. Although $30 million per year by 2004 is required to meet the business plan, $15 million was raised for 1999/2000. MMV has received support from development agencies, foundations, industry, and health agencies. The organization is now selecting a second round of projects after a promising selection of three first-round projects from 101 applications.

Besides public/private partnerships to develop new drugs, some groups have programs to control infectious diseases in the developing world using donations of existing drugs. Industry has made substantial donations to programs against onchocerciasis, lymphatic filariasis, drug-resistant malaria, trachoma, and leprosy that involve donations of Mectizan, Albendazole, Malarone, Zithromax, and Leprosy MDT, respectively. Sixty percent of corporate contributions to philanthropy were product donations (3). In all cases the contribution from the company has gone far beyond the provision of the drug to supporting development of systems that will ensure the efficient distribution and effective use of donated drugs.

The incentive for this kind of project is altruism, which has obvious limits in the competitive commercial environments. Ventures that provide both "push" and "pull" interventions are more likely to be sustained. A company or partnership underwriting the cost of research and development is a push intervention. Creating a market for the drugs or vaccines being developed is a pull intervention. MMV uses both strategies, supporting drug development and working through WHO's Roll Back Malaria activity and other partners to assure the existence of a market for the drugs.

### Partnership for Policy Development: Institute of Medicine Emerging Infections Activities

In May 1989, Rockefeller University, the National Institute of Allergy and Infectious Diseases, and the Fogarty International Center cosponsored a conference on emerging viral agents. Although the conference focused on viruses, it spurred interest in the emergence and resurgence of all classes of infectious agents. Subsequently, the Institute of Medicine (IOM) convened a panel and carried out a study under the leadership of Joshua Lederberg and Robert Shope that resulted in the 1992 report Emerging Infections: Microbial Threats to Health in the United States (4). Funding for this study was provided by the Centers for Disease Control and Prevention (CDC), the Fogarty International Center, Lederle-Praxis Laboratories, the Lucille B. Markey Charitable Trust, the National Institute of Allergy and Infectious Diseases, the Rockefeller Foundation, and the U.S. Army Medical Research and Development Command. From the beginning, the broad support from private and public agencies provided the foundation for an ongoing partnership for policy development.

The report called for increasing investments in the public health infrastructure, especially in surveillance, research, and training; in the development and deployment of vaccines and antimicrobial drugs and the control of resistance; vector control; and research on personal and community health practices relevant to disease transmission. Perhaps the most effective response to the report came CDC under the leadership of Walter Dowdle, James Hughes, and Ruth Berkelman, who put together a CDC plan for addressing the issues raised in the report (5). This plan galvanized congressional attention, and with the advocacy of groups like the American Society for Microbiology and others, drew attention to the need for additional resources and investments in treating and preventing emerging infections. Subsequently, in partnership CDC and NIAID asked the IOM to establish the Forum on Emerging Infections as a convening ground for public and private agencies to address continuing issues and problems related to emerging infections. The Burroughs Wellcome Fund also supports this activity in which policy makers can address the problem of emerging infections.

### Philanthropic Investments

What role can philanthropy play in public/private partnerships to address emerging infections? Foundations are uniquely qualified to initiate thought and action, experiment with new and untried ventures, dissent from prevailing attitudes, and act quickly and flexibly (6). Several kinds of foundations are common. Independent foundations, such as the Bill and Melinda Gates Foundation, are usually established by an individual. Company-sponsored foundations include the

Merck Foundation. The American Cancer Society is an example of an operating or special interest foundation. The Research Triangle Community Foundation is a community foundation, which usually raise and manage money from different donors and direct the contributions locally.

Foundation type as much as size influences patterns of giving and growth. In 1999 there were 47,000 foundations in the United States, many of them small family foundations with assets of about $1 million. In the United States, foundations with assets of $50 million or more represent 2% of foundations, yet control 71% of total assets (7). In 1999, total giving in the United States amounted to $190.16 billion (8). Most contributions, nearly $160 billion, came from individual donations; foundations contribute nearly $20 billion, and corporations and their foundations more than $11 billion (60% in the form of product donations). Health care received only 9.4% of philanthropic donations (Figure 1) (8). In contrast, in 1996 U.S. health research and development expenditures were close to $38 billion, with industry investing more than half of those dollars and the foundation contributions amounting to only 4% of the total (Figure 2) (9). A 1997 survey of private funders of biomedical research in the United States showed that $1.3 billion was invested that year. Thus philanthropic support of $1-2 billion (10) for medical research is small in comparison with that from NIH (1999 budget $15.6 billion) or that from industry ($22 billion).

The entry of the Bill and Melinda Gates Foundation in support of international health R&D has had a stunning effect because of the comparatively large amount of money it has committed to the philanthropically undervalued area of international health. Perhaps, other foundations could be recruited to support this area during the anticipated transfer of wealth projected over the next decade if the public health community makes a cogent case for the need and value of such investment.

Philanthropic organizations can move quickly to fill a gap, function as neutral conveners, model successful approaches, develop information for policy debate, fund politically unpopular areas of research, and take risks. Drawbacks of philanthropic support include limited funds for research, less willingness to support overhead or infrastructure, the desire to model programs and move on, and the tendency to resist collaborative ventures. Thus, philanthropic organizations can be catalysts for developing public/private partnerships, but these groups do have limitations because they cannot commit as much money to emerging infections research as industry or government agencies.

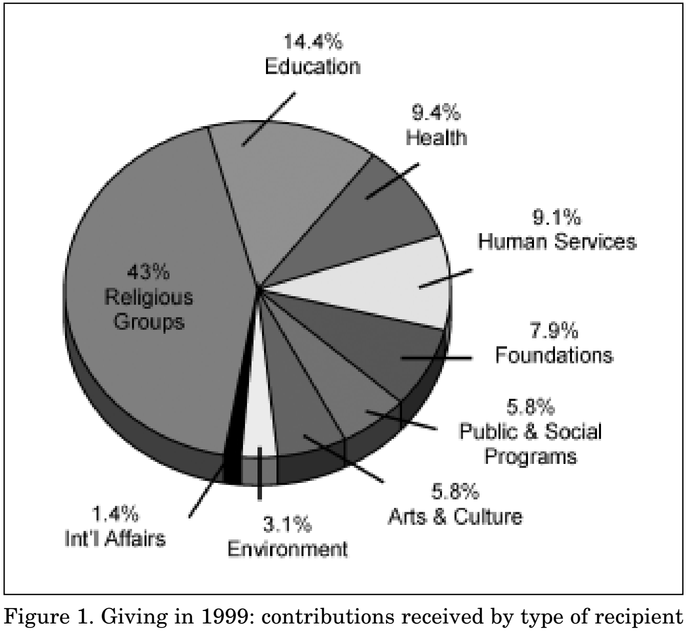

Figure 1. Giving in 1999: contributions received by type of recipient organization in the United States (8)

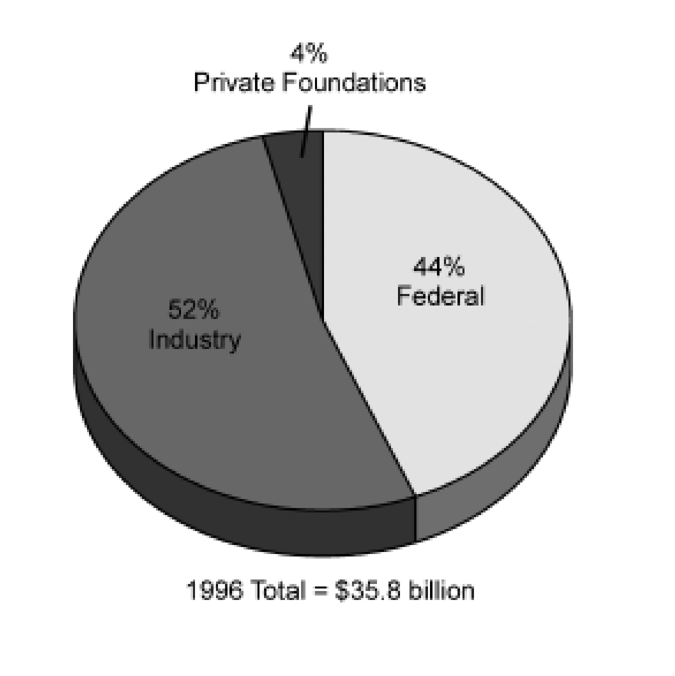

Figure 2. U.S. health research and development expenditures (9)

### Conclusions

The following lessons can be derived from these examples of public/private partnership and an examination of philanthropic capacity:

1. Philanthopic support, though important as risk capital in the system, is modest in comparison to industrial and government support for medical research.
2. The amount of wealth expected to be transferred during the period 1990-2040 has been estimated to exceed $10 trillion (11), thus providing opportunities to capture additional dollars for medical/health research and international health.
3. Industry is an essential partner but needs both “push” and “pull” mechanisms to participate in drug and vaccine development for diseases that largely affect poor people.
4. Public health and government agencies need long-term, increased investments to advance knowledge, to develop vaccines and drugs, and to control emerging infectious diseases.
5. Owing to the complexity and global nature of the issues in emerging infections, partnerships are more important today than ever before.

Dr. Bond is president of the Burroughs Wellcome Fund. Before 1994, she was the executive officer of the Institute of Medicine. Dr. Bond currently serves as the chair of the National Center for Infectious Diseases Board of Scientific Counselors.

### References

1. Institute of Medicine. America's vital interest in global health. Washington: National Academy Press; 1997.
2. Hoffman S. Research (genomics) is crucial to attacking malaria. Science 2000;290:1509.
3. Lindquist D. Drug companies' Rx for the bottom line (charitable giving). Industry Week 7 Sept 1998;247:1.
4. Lederberg J, Shope R, Oaks S Jr, editors. Emerging infections: microbial threats to health in the United States. Washington: National Academy Press; 1992.
5. Centers for Disease Control and Prevention. Addressing emerging infectious disease threats: a prevention strategy for the United States. Atlanta: Public Health Service, U.S. Department of Health and Human Services; 1994.
6. Treasury Department Report on Private Foundations: Committee on Finance of the United States Senate, 89th Cong., 1st Sess. (1965).
7. Lawrence S, Camposeco C, Kendzion J. Foundation giving trends: update on funding priorities. New York: The Foundation Center; 2000.
8. American Association of Fund-Raising Counsel. Giving USA 2000. New York: AAFRC Trust for Philanthropy; 2000.
9. Heinig A, Quon A, Meyer R, Korn D. The changing landscape for clinical research. Acad Med 1999;74:725-45.
10. American Cancer Society, Burroughs Wellcome Fund, Howard Hughes Medical Institute, The Pew Charitable Trusts. Strengthening health research in America: philanthropy's role. Washington: The Foundations; 1998.
11. Gergen D. I hereby bequeath: coming soon, a $10 trillion chance to build American philanthropy. U.S. News and World Report 28 April 1997;122:80.
